# Supervised learning with decision margins in pools of spiking neurons

**DOI:** 10.1007/s10827-014-0505-9

**Published:** 2014-05-28

**Authors:** Charlotte Le Mouel, Kenneth D. Harris, Pierre Yger

**Affiliations:** 1 UCL Institute of Neurology and UCL Department of Neuroscience, Physiology, and Pharmacology, London, UK; 2 Ecole Normale Supérieure, Paris, France

**Keywords:** Keywords, Supervised learning, Spiking neurons, Tempotron, Support vector machine

## Abstract

Learning to categorise sensory inputs by generalising from a few examples whose category is precisely known is a crucial step for the brain to produce appropriate behavioural responses. At the neuronal level, this may be performed by adaptation of synaptic weights under the influence of a training signal, in order to group spiking patterns impinging on the neuron. Here we describe a framework that allows spiking neurons to perform such “supervised learning”, using principles similar to the Support Vector Machine, a well-established and robust classifier. Using a hinge-loss error function, we show that requesting a margin similar to that of the SVM improves performance on linearly non-separable problems. Moreover, we show that using pools of neurons to discriminate categories can also increase the performance by sharing the load among neurons.

**Electronic supplementary material:**

The online version of this article (doi:10.1007/s10827-014-0505-9) contains supplementary material, which is available to authorized users.

## Introduction

To make sense of the world, animals must distinguish the sensory input patterns that characterize different objects or situations. In some cases, specific sensory patterns have innate behavioural associations, such as the species-typical meanings of animal vocalizations, for example growls and whines (Altenmüller et al., 2013). In other cases however, these associations must be learned. In the laboratory, pairing an initially-neutral conditioned stimulus such as a tone, light, or odour with an aversive unconditioned stimulus such as a foot shock leads an animal to respond similarly to the conditioned as to the unconditioned stimulus; such learning is believed to depend on synaptic plasticity in the amygdala (Pape & Pare, 2010). Repeated performance of an action in a given circumstance leads to the formation of stimulus–response associations or habits, which are believed to develop through synaptic plasticity in the dorsal striatum (Yin & Knowlton, 2006). Importantly, learning of stimulus categories does not require any explicit behaviour, reward or punishment. For example, new born female Belding’s ground squirrels learn the odours of their siblings simply by their presence in the nest during early life; this association allows later identification of kin during adulthood (Holmes, 1986).

In statistics and machine learning, association of input patterns with desired categories, as specified by a training signal, is referred to as supervised learning. This form of learning should be distinguished from reinforcement learning, in which learning is governed by a reward rather than an explicit training signal; and unsupervised learning, in which representations are found based on structure in the input, without any explicit training signal. A classical algorithm for supervised learning is the Perceptron learning rule F. Rosenblatt 1958), which trains a single artificial neuron to linearly weight its inputs such that category is predicted by whether the weighted sum exceeds a fixed threshold. The Support Vector Machine (SVM) improves on perceptron performance by using a *margin* (a gap between the training boundaries for different classes), as well as through other innovations such as the introduction of nonlinearities through a kernel function (Cortes & Vapnik, 1995).

A number of learning rules have been suggested by which spiking neurons might perform tasks analogous to supervised learning (Bohte et al., 2002; Florian, 2007; Pfister et al., 2006; Ponulak & Kasiński, 2010; Xu et al., 2013; Legenstein et al., 2005). Recently, concepts of the Perceptron were extended to spiking neurons in a framework called the “Tempotron”, in which an error signal is used to adjust synapses strongly active when the neuron was close to its threshold (Florian, 2012; Gutig & Sompolinsky 2006; Gütig & Sompolinsky, 2009), producing 1 or 0 spikes according to the desired category. In the present work, we describe an adaptation of the SVM to spiking neurons, whose margin allows for the training of more general firing rate modulations than 0/1 spike. We found that a moderate training margin increases the learning speed of single neurons in linearly separable tasks, and increases their performance in linearly non-separable tasks. To further improve learning of linearly non-separable problems, we considered an extension in which neurons work in pools trained simultaneously (Urbanczik & Senn, 2009), whose combined activity forms the network’s response to a pattern. We found that this indeed improved performance as the training signal, although global, nevertheless allowed different neurons to learn different receptive fields.

## Material and methods

### Neuron model

In all simulations, we used a conductance-based integrate-and-fire neuron model with a membrane time constant τ_m_ = 20ms, a leak conductance g_L_ = 10nS, and a resting membrane potential V_rest_ = − 70mV. Spikes were generated when the membrane potential V_m_ reached the threshold V_thresh_ = − 50mV. To model the shape of the action potential, the voltage was set to 20 mV after threshold crossing, and then decayed linearly during a refractory period of duration τ_width_ = 5ms to the reset value V_reset_ = − 55mV, following which an exponentially decaying depolarizing current of initial magnitude 50pA and time constant τ_dep_ = 40ms was applied (similarly to (Clopath et al., 2010; Yger & Harris, 2013)). We used this scheme with a high reset voltage and ADP, rather than the more common low reset value, as it provides a better match to intracellular recordings *in vitro* and *in vivo*. Synaptic connections were modelled as transient conductance changes with instantaneous rise followed by exponential decay. Synaptic connections were excitatory only (synaptic weights were clipped when they attempted to cross zero), with a time constant τ_exc_ = 5ms and a reversal potential E_exc_ = 0mV.

### Input patterns

To reduce the time of the simulations, we used only 10 input neurons. For each input pattern, the firing rate of each input neuron is independently drawn from a uniform distribution between 0 and 1Hz. The rate pattern is then normalised such that the total input rate is 10 000Hz, comparable to the physiological regime in which neurons operate (assuming an average of 10,000 incoming synapses at 1Hz). Every time a pattern is presented, the rate pattern is transformed into a novel 100 ms spiking pattern via the realization of ten independent and homogeneous Poisson processes.

### Network structure

**Fig. 1:**
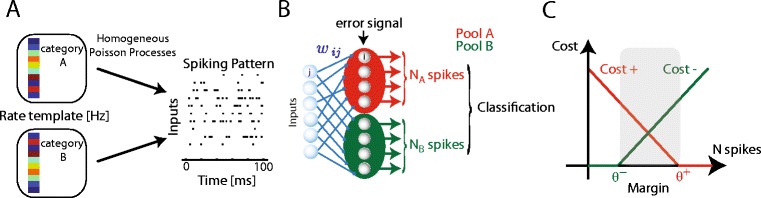
Illustration of the classification task. (**a**), Random normalized rate patterns are drawn and assigned to two categories, (**a**) and (**b**). Every time a pattern is presented, the rate pattern is transformed into a novel 100 ms spiking pattern via a homogeneous Poisson process. (**b**), Schematic of the network used for classification. There is an all-to-all connection between the input neurons and the neurons in pools (**a**) and (**b**), and no lateral connections or inhibition. The cost function for each pool is calculated based on the input’s category and the number of spikes emitted by that pool; the error signal is then distributed to all neurons in that pool. Classification is based on comparison between the two pools’ numbers of spikes N_A_ and N_B_. (**c**) The cost function for each pool, adapted from the SVM, as a function of the number of spikes N_spikes_ emitted by the pool: if the input belonged to the pool’s category (red curve) it is the number of spikes missing to reach θ_+_; otherwise (green curve), it is the number of spikes exceeding θ_−_

Input spike trains are fed, in an all-to-all manner, to neurons embedded in two pools, termed A and B (see Fig. 1). There are either one or several (three) neurons in each pool, and connections are established with initial weights drawn from a uniform distribution between 0 and 2nS, and delays drawn from a uniform distribution between [0.1 ms, 5 ms]. There are no lateral connections between the neurons in the pools.

### Learning rule

We derived the learning rule from approximate gradient descent on the Support Vector Machine cost function (see Fig. 1 panel C). This cost function *E* for a neuronal pool on a given trial is a function of the summed number of spikes N_pool_ emitted by all the neurons within the pool during that trial, and of the category of the input pattern presented during that trial. This function depends on two parameters, the learning thresholds θ_+_ and θ_−_. If the input pattern belongs to the same category as the pool, then the pool should respond to it by emitting at least θ_+_ θ_+_ spikes. If this is the case then the cost for the pool is 0, otherwise it is equal to the number of missing spikes. The cost function is thus a rectified linear function of the pool’s number of spikes, with parameter θ_+_. If the input pattern is not of the same category as the pool, then the pool should respond to it by emitting less than θ_−_ spikes. If this is the case then the cost for the pool is 0, otherwise it is equal to the number of superfluous spikes. The cost function is thus a rectified linear function of the pool’s number of spikes, with parameter θ_−_.

To perform gradient descent on the synaptic weights, the change in synaptic weight w_ij_ from the input neuron *j* to the neuron *i* of the pool must be proportional to the opposite of the derivative of this cost function with respect to w_ij_. Due to Poisson noise in the inputs, for a given set of weights, the response to a given pattern may vary from trial to trial. We therefore consider the derivative of the expected cost function. With the chain rule, this is equal to the product of the derivative of the expected cost *E* with respect to the expected summed number of spikes of the pool < N_pool_ > (because of the hinge-loss function, this derivative takes the values −1, 0 or 1); of the derivative of < N_pool_ > with respect to the expected number of spikes < n_i_ > of neuron *i* (this derivative takes the value 1 expect at < n_i_ > = 0, where it is set to 0 or 1 so as to incorporate the constraint that neurons which do not spike are not allowed to reduce their incoming synaptic weights but can increase them); and of the derivative of < n_i_ > with respect to w_ij_. This leads to the following equation for the weight update:

1 $$ {\Delta \mathrm{w}}_{\mathrm{i}\mathrm{j}}\propto -\frac{\mathrm{d}\mathrm{E}}{\mathrm{d}<{\mathrm{N}}_{\mathrm{pool}}>}\frac{\mathrm{d}<{\mathrm{N}}_{\mathrm{pool}}>}{\mathrm{d}<{\mathrm{n}}_{\mathrm{i}}>}\frac{\mathrm{d}<{\mathrm{n}}_{\mathrm{i}}>}{{\mathrm{d}\mathrm{w}}_{\mathrm{i}\mathrm{j}}} $$

We refer to the last term as the eligibility trace. To obtain it, we first approximate n_i_ by a function of the square of the membrane voltage V_i_ of neuron *i*:

2 $$ <{\mathrm{n}}_{\mathrm{i}}>\propto {\displaystyle {\int}_0^T}{\mathrm{F}}^2\left({\mathrm{V}}_{\mathrm{i}}\left(\mathrm{t}\right)\right)\mathrm{dt} $$

*T* being the length of a trial, and *F* a rectified linear function of the voltage:

3 $$ \mathrm{F}\left(\mathrm{V}\right)=0\ \mathrm{if}\ \mathrm{V}<-60 mV $$

4 $$ \mathrm{F}\left(\mathrm{V}\right)=60\mathrm{mV}+\mathrm{V}\ \mathrm{if}\ \mathrm{V}>-60\mathrm{mV} $$

This approximation captures well the relationship between firing rate and voltage in a typical trial generated with the input statistics of the classification task (Supplementary Figure 1).

Assuming this, we then have:

5 $$ \frac{\mathrm{d}<{\mathrm{n}}_{\mathrm{i}}>}{{\mathrm{d}\mathrm{w}}_{\mathrm{i}\mathrm{j}}}\propto {\displaystyle {\int}_0^T}\mathrm{F}\left({\mathrm{V}}_{\mathrm{i}}\left(\mathrm{t}\right)\right)\frac{{\mathrm{d}\mathrm{V}}_{\mathrm{i}}\left(\mathrm{t}\right)}{{\mathrm{d}\mathrm{w}}_{\mathrm{i}\mathrm{j}}}\mathrm{dt} $$

We estimate $\frac{{\mathrm{dV}}_{\mathrm{i}}\left(\mathrm{t}\right)}{{\mathrm{dw}}_{\mathrm{i}\mathrm{j}}}$ similarly to the method of (Gutig & Sompolinsky 2006; Gütig & Sompolinsky, 2009). For a conductance-based integrate-and-fire neuron as used in this study, ignoring the reset mechanism due to a spike, we have:

6 $$ {\mathrm{c}}_{\mathrm{m}}\frac{{\mathrm{dV}}_{\mathrm{i}}\left(\mathrm{t}\right)}{\mathrm{dt}}=-\left({\mathrm{V}}_{\mathrm{i}}\left(\mathrm{t}\right)-{\mathrm{V}}_{\mathrm{rest}}\right)\left({\mathrm{g}}_{\mathrm{L}}+{\mathrm{G}}_{\mathrm{exc}}{\left(\mathrm{t}\right)}_i\right)+{\mathrm{I}}_{\mathrm{syn}}{\left(\mathrm{t}\right)}_i $$

where G_exc_(t)_*i*_ is the total synaptic excitatory conductance and I_syn_(t)_*i*_ the synaptic current of neuron *i*. Specifically, if ${t}^j,\dots, {t}_{N_j}^j$ are the *N*_*j*_ times at which a particular synapse *j* of weight w_ij_ is active, if $\mathrm{g}\left(\mathrm{t}\right)={\mathrm{e}}^{-\mathrm{t}/{\uptau}_{\mathrm{syn}}}$ (if t > 0) is the kernel function representing the conductance time course, and if *N* is the number of input synapses (here 10), we have:

7 $$ {\mathrm{G}}_{\mathrm{exc}}{\left(\mathrm{t}\right)}_i={\displaystyle \sum_{\mathrm{j}=1}^{\mathrm{N}}}{\mathrm{w}}_{\mathrm{ij}}{\displaystyle \sum_{\mathrm{s}=1}^{{\mathrm{N}}_{\mathrm{j}}}}\mathrm{g}\left(\mathrm{t}-{t}_s^j\right)\ \mathrm{and}\ {\mathrm{I}}_{\mathrm{s}\mathrm{yn}}{\left(\mathrm{t}\right)}_i=\left({\mathrm{E}}_{\mathrm{exc}}-{\mathrm{V}}_{\mathrm{rest}}\right){\displaystyle \sum_{\mathrm{j}=1}^{\mathrm{N}}}{\mathrm{w}}_{\mathrm{ij}}{\displaystyle \sum_{\mathrm{s}=1}^{{\mathrm{N}}_{\mathrm{j}}}}\mathrm{g}\left(\mathrm{t}-{t}_s^j\right) $$

Inspecting former equations, we see that for a conductance-based neuron, V_i_ integrates I_syn_(t)_*i*_ with an effective time constant τ_eff_ = c_m_/(g_L_ + G_exc_(t)_*i*_). Approximating τ_eff_ by a constant equal to c_m_/(g_L_ + < G_exc_(t)_*i*_ >) where < G_exc_(t)_*i*_ > denotes a running average of the synaptic conductance during the presentation of one pattern (Gütig & Sompolinsky, 2009), we can approximate V_i_(t) by the following equation:

8 $$ {\mathrm{V}}_{\mathrm{i}}\left(\mathrm{t}\right)\approx {\displaystyle \sum_{\mathrm{j}=1}^{\mathrm{N}}}\frac{w_{ij}}{g_L}\left({\mathrm{E}}_{\mathrm{exc}}-{\mathrm{V}}_{\mathrm{rest}}\right){\displaystyle \sum_{\mathrm{s}=1}^{{\mathrm{N}}_{\mathrm{j}}}}\mathrm{K}\left(\mathrm{t}-{t}_s^j\right)+{\mathrm{V}}_{\mathrm{rest}} $$

Where input spikes evoke PSPs of shape $$ \mathrm{K}\left(\mathrm{t}\right)=\frac{\left({e}^{-\raisebox{1ex}{$t$}\!\left/ \!\raisebox{-1ex}{${\tau}_{syn}$}\right.}-{e}^{-\raisebox{1ex}{$t$}\!\left/ \!\raisebox{-1ex}{${\tau}_{eff}$}\right.}\right)}{\frac{\tau_m}{\tau_{eff}}-\frac{\tau_m}{\tau_{syn}}} $$. Therefore, we approximate the derivative of the post-synaptic potential $$ \frac{{\mathrm{dV}}_{\mathrm{i}}\left(\mathrm{t}\right)}{{\mathrm{dw}}_{\mathrm{i}\mathrm{j}}} $$ by a sum of PSPs (whose height and time-course are fixed for a given trial) at times $$ {t}_1^j,\dots, {t}_{N_j}^j $$ when the input neuron *j* spiked:

9 $$ \frac{{\mathrm{dV}}_{\mathrm{i}}\left(\mathrm{t}\right)}{{\mathrm{dw}}_{\mathrm{i}\mathrm{j}}}\propto \left({\mathrm{E}}_{\mathrm{exc}}-{\mathrm{V}}_{\mathrm{rest}}\right){\displaystyle \sum_{\mathrm{s}=1}^{{\mathrm{N}}_{\mathrm{j}}}}\mathrm{K}\left(\mathrm{t}-{t}_s^j\right) $$

Ignoring the reset mechanism and the non-linearity due to the spike, this would be exact for a current based neuron, but this is only an approximation for the conductance-based neuron which we implement, estimating the average membrane time constant with the average conductance received during the presentation of a single pattern (Gütig & Sompolinsky, 2009).

We therefore obtain the following learning rule:

10 $$ {\Delta \mathrm{w}}_{\mathrm{i}\mathrm{j}}\propto -\frac{\mathrm{d}\mathrm{E}}{\mathrm{d}<{\mathrm{N}}_{\mathrm{pool}}>}\frac{\mathrm{d}<{\mathrm{N}}_{\mathrm{pool}}>}{\mathrm{d}<{\mathrm{n}}_{\mathrm{i}}>}{\displaystyle \underset{0}{\overset{T}{\int }}}\mathrm{F}\left({\mathrm{V}}_{\mathrm{i}}\left(\mathrm{t}\right)\right){\displaystyle \sum_{\mathrm{s}=1}^{{\mathrm{N}}_{\mathrm{j}}}}\mathrm{K}\left(\mathrm{t}-{t}_s^j\right)\mathrm{dt} $$

We impose the constraint that weights that attempt to become negative are clipped to zero, since we are using only excitatory synapses. In addition, we add a constraint that a neuron that doesn’t fire cannot reduce its incoming synaptic weights.

### Neural simulator

Simulations of the spiking neurons were performed using a customised version of the NEST simulator (Diesmann & Gewaltig, 2007) and the PyNN interface (Davison et al., 2009), with a fixed time step of 0.1 ms.

### Support vector machine

For Figs. 5, 10, the linear Support Vector Machine of the Python scikit toolkit (Pedregosa & Varoquaux, 2011) was trained on Poisson spike counts drawn from the same patterns that were used to train the neuronal pools. For each pattern number, the cost parameter (termed *c*) was chosen so as to optimise the SVM performance. This yielded the same optimal cost parameter c = 10^− 6^ for all pattern numbers. In Fig. 5, performance for a lower and a higher value of the cost parameter are also shown.

## Results

We studied a learning algorithm for spiking neurons to perform supervised learning, based on the support vector machine (SVM) cost function. The network that was used for the task is shown in Fig. 1a. Working in a rate-based framework, we defined each input pattern by a set of mean rates of each of the input neurons during that pattern, which is transformed into a 100 ms spiking pattern via a homogeneous Poisson process generated anew each time a pattern is presented (see Fig. 1a left, and Material and Methods). The input patterns are normalised random rate vectors (randomly assigned to the two categories A and B, see Materials and Methods). Fig. 1a is a schematic illustration of the learning task addressed by the spiking neurons, and of the generation process of the input spike trains from the input patterns of the two categories. All neurons in the two readout pools received connections from all input neurons. There are no lateral connections between the readout neurons (Fig. 1b). Each pool is assigned one category of inputs to which it must respond, its positive (+) patterns; the other patterns become the pool’s negative (−) patterns. Pool A’s + patterns are thus the A patterns, while its patterns are the B patterns. Classification is assessed as correct if in response to an A pattern, the summed number of spikes from pool A, N_A_ is greater than the summed number of spikes from pool B, N_B_ (and vice versa).

The learning rule was designed by adapting the SVM cost function to spiking neurons, using the framework of the Tempotron learning rule (see Materials and Methods). The neurons in a pool are trained to emit collectively at least θ_+_ spikes to their + patterns and less than θ_−_ to their patterns using a cost function which counts the number of missing or superfluous spikes (illustrated in Fig. 1c). The “hinge” shape of this cost function is directly inspired from SVM techniques (Cortes & Vapnik, 1995). On the trials in which a pool emits an incorrect number of spikes (less than θ_+_ in response to a + pattern, or more than θ_−_ in response to a pattern), it receives an error signal indicating whether it has fired too many or too little spikes, allowing it to perform approximate gradient descent on this cost function, ensuring that after each update the cost is decreased (for the complete derivation see the Material and Methods section). The rule obtained has the form:

11 $$ {\Delta \mathrm{w}}_{\mathrm{i}\mathrm{j}}\propto -\frac{\mathrm{d}\mathrm{E}}{\mathrm{d}<{\mathrm{N}}_{\mathrm{pool}}>}\frac{\mathrm{d}<{\mathrm{N}}_{\mathrm{pool}}>}{\mathrm{d}<{\mathrm{n}}_{\mathrm{i}}>}{\displaystyle \underset{0}{\overset{T}{\int }}}\mathrm{F}\left({\mathrm{V}}_{\mathrm{i}}\left(\mathrm{t}\right)\right){\displaystyle \sum_{t_{s\in \left\{1,..,,{\mathrm{N}}_{\mathrm{j}}\right\}}^j}}\mathrm{K}\left(\mathrm{t}-{t}_s^j\right)\mathrm{dt} $$

where *K* can be seen as a normalized EPSP at the soma, *F* is a rectified linear function, *N*_*pool*_ is the total number of spikes emitted by one pool, and n_i_ is the number of spikes emitted by neuron *i*. The supervision or error signal is defined as:

12 $$ \frac{\mathrm{d}\mathrm{E}}{\mathrm{d}<{\mathrm{N}}_{\mathrm{pool}}>}=\left\{\begin{array}{c}\hfill 0\  if\ \left(+ pattern\  and\ {N}_{pool}\ge {\uptheta}_{+}\right)\  or\  if\ \left(- pattern\  and\ {N}_{pool}\le {\uptheta}_{-}\right)\ \hfill \\ {}\hfill -1\  if\ \left(- pattern\  and\ {N}_{pool}>{\uptheta}_{-}\right)\hfill \\ {}\hfill +1\  if\ \left(+ pattern\  and\ {N}_{pool}<{\uptheta}_{+}\right)\hfill \end{array}\right. $$

In our learning rule, each synapse thus accumulates an eligibility trace over the course of a trial. At the end of each trial, if the neuron receives an error signal, the eligibility trace is transformed into a synaptic change, the sign of which is dictated by the error signal. This defines a 3-factor learning rule: if there is no error signal, no plasticity occurs; if the error signal is positive, the rule is Hebbian (inputs that make the neuron fire are potentiated); but if the error signal is negative, the rule is anti-Hebbian (inputs that make the neuron fire are depressed). Therefore, unlike purely Hebbian or STDP rules that require homeostasis to ensure stability (Abbott & Nelson, 2000; Clopath et al., 2010; Yger & Harris, 2013), this rule is intrinsically stable. Note that such a notion of eligibility traces has already been proposed in the case of reinforcement learning with a delayed error signal (Izhikevich, 2007; Legenstein et al., 2008).

**Fig. 2:**
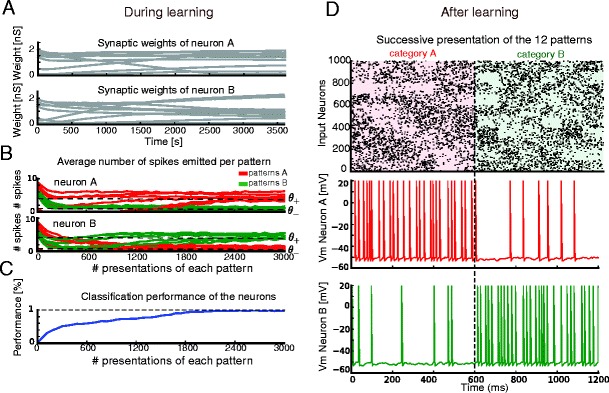
Example of single neuron pools learning to classify 12 patterns with learning thresholds (θ_−,_θ_+_) = (1, 4). A, Evolution of the ten synaptic weights incoming onto neuron (**a**) (top) and neuron (**b**) (bottom), as a function of time during learning. B, Evolution of the number of spikes produced by neuron (**a**) (top) and (**b**) (bottom) in response to patterns of category (**a**) (red curves) and (**b**) (green curves), as a function of the number of presentations of each pattern. C, Evolution of the classification performance as a function of time, reaching 100 % after learning. Generalization performance was evaluated by using different data samples than those used to train the classifier. D, Successive presentation of the six patterns of category (**a**) followed by the six patterns of category (**b**). (top) Schematic of the input spike trains: the spikes of each of the ten input synapses are spread out over 100 synapses for illustrative purposes. Voltage traces after learning for neuron (**a**) (middle) and (**b**) (bottom). After learning, each neuron reliably spikes to the inputs from its category, with nonzero baseline firing

As a first example, we trained two single neurons to classify 12 random rate patterns with 10 input synapses and learning thresholds θ_+_ = 4 and θ_−_ = 1. Synaptic weights evolved throughout learning (see Fig. 2a; a 1 ns conductance gives rise to 0.5 mV EPSP in the fluctuation conditions of a typical trial), and after training, each neuron fired at least 3 spikes to each of the patterns of its category and at most 2 spikes to each of the other patterns (Fig. 2b) leading to an almost perfect classification (Fig. 2c). Fig. 2d illustrates the responses after learning to the six “A” patterns immediately followed by the six “B” patterns. The neuron from pool A was strongly active during the first 6 patterns, while the one from pool B was active during the latter 6. The readout neurons thus reliably spiked to their categories. We verified that learning behaviour was not affected by the number of input synapses (Supplementary Figure 2). For the remainder of the text, we therefore used 10 input synapses.

**Fig. 3:**
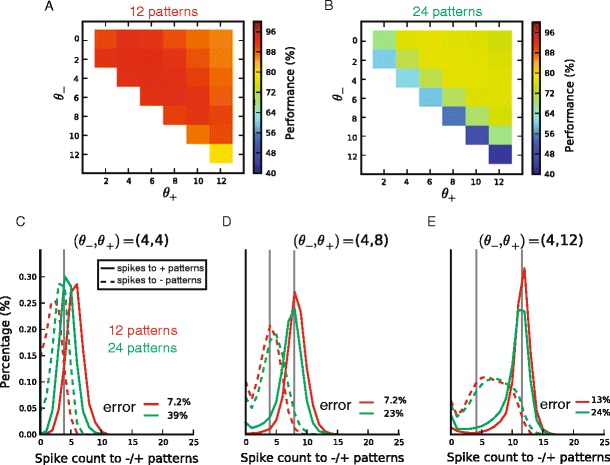
Generalization performance of single neuron pools as a function of the training margin M = θ_+_ − θ_−_. Performance as a function of the learning thresholds θ_+_ and θ_−_ for the classification of (**a**), 12 input patterns, for which performance is highest for low values of the margin (near the diagonal); and (**b**), 24 input patterns, for which performance is highest for larger values of the margin (off the diagonal). C, (**d**), (**e**) Histograms of the number of spikes emitted by the neurons in response to inputs from their category (+patterns, plain curves) and from the other category (patterns, dotted lines), in the classification of 12 patterns (red curves), and 24 patterns (green curves), and for different values of the training margin M = θ_+_ − θ_−_, respectively *M* = 0, (θ_−,_θ_+_) = (4, 4) (panel (**c**)) *M* = 4, (θ_−,_θ_+_) = (4, 8) (panel (**d**)), and *M* = 8, (θ_−,_θ_+_) = (4, 12) (panel E). Classification errors are written in the bottom right of the panels. The histograms to −/+patterns are wider for larger values of the margin, and are drawn closer when the number of patterns increases; both of which lead to a higher overlap and therefore a poorer performance

We then asked how learning depends on the margin M = θ_+_ − θ_−_ between the learning thresholds. To answer this question, we performed the same task, i.e. the classification of 12 random rate patterns, but with different values of the learning thresholds (θ_−_, θ_+_) ranging from 0 to 12. The classification performance after learning is plotted as a function of the learning thresholds in Fig. 3a. As one can see, performance on the nearly separable 12-pattern task was better with a smaller margin, decreasing when  M  exceeds 4 spikes. However, when the task was made more complex using 24 input patterns (which is a highly linearly non-separable task in 10 dimensions), a clear benefit of the margin was seen (see bad performance on the diagonal where  M = 0  in Fig. 3b).

The fact that optimal performance could be obtained in the 12 pattern case with a margin of 0 was at first surprising. For example, when trained with equal thresholds (θ_−_, θ_+_) = (4, 4), if each pool emitted exactly 4 spikes to every pattern, they would receive no error signal during training, yet their classification performance would be 0 %. To investigate how good performance could be obtained without a margin in a close-to-linearly separable situation, we plotted a histogram of spike count outputs (see Fig. 3c). Note that the spike count distribution of each category is broad and bell-shaped, even after learning; this reflects the random distribution of the multiple patterns in each class. High classification without a margin occurred because the centres of the distributions are widely separated. This can be characterised by the difference between the mean spike count in response to target patterns and the mean spike count in response to null patterns, which we will refer to as spike count modulation. We suggest that modulation occurs because the hinge cost function causes plasticity anytime the response exceeds the learning threshold, and the broadness of the spike count distributions for each class causes the centres of the spike count histograms to move apart, resulting in spike count modulation even when no margin was requested. For 24 patterns however, this separation did not occur, suggesting that in a highly nonlinearly separable problem, spike count modulation only occurs when a margin is explicitly requested.

We next asked how requesting a margin affected performance in the two cases. Figs. 3c damd e show histograms, for various margins, of the spike counts emitted by each neuron in response to its + patterns (full lines) and in response to its patterns (dashed lines). As the margin is increased, the spike distributions move further apart, allowing better separation in the case of 24 patterns (green). For 12 patterns however (red), because separation already occurred without a margin, little gain was derived from the margin, and indeed performance actually decreased in the case of an 8-spikes margin, likely due to the broadening of the response distribution for patterns. We speculate this may occur because in order to respond very strongly to + patterns, the neurons cannot avoid also producing strong responses to at least some patterns.

**Fig. 4:**
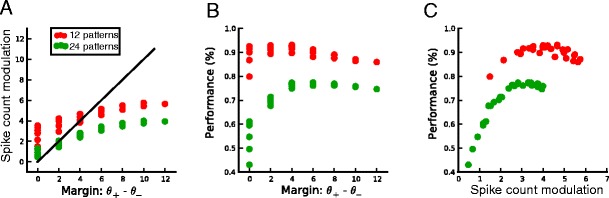
Relationship between training margin and actual spike count modulation for single-neuron pools (defined as the difference between the mean spike count in response to target patterns and the mean spike count in response to null patterns). A, Scatterplot of the spike count modulation after learning as a function of the training margin. The actual modulation does not track the training margin, and is systematically larger for easier tasks (12 patterns in red versus 24 patterns in green) (**b**), Scatter plot of the performance as a function of the training margin; there is a wide spread for low values of the margin. C, Same as in (**b**), but as a function of the spike count modulation; the relationship is more tightly constrained

Based on these results, we speculated that the critical parameter determining performance is the actual separation between the spike count histograms for + and patterns, rather than the margin requested. Figure 4a shows the actual spike count modulation as a function of the margin M = (θ_+_ − θ_−_) for 12 patterns (red) and 24 patterns (green) to classify. The spike count modulation does not track the margin, as seen by the shallow slope of this curve; in addition it is systematically bigger when the task is easier (12 versus 24 patterns to classify). This confirms the intuitive explanation for how a margin of zero can give a variety of performances (Fig. 4b), whereas the relationship between spike count modulation and performance (Fig. 4c) is much tighter and much more constrained.

**Fig. 5:**
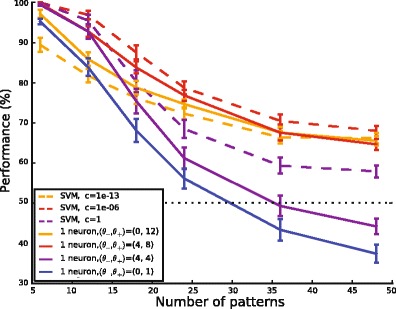
Comparison of the performance of single-neuron pools and a linear SVM. Performance of a linear SVM (dashed lines) and a single neuron (full lines) as a function of the number of patterns to be learned. When the training parameter places a high emphasis on the margin (yellow, margin = 12 for the neuron, c = 10^− 13^ for the SVM) performance is suboptimal for low numbers of patterns. When the training parameter places a low emphasis on the margin (purple, margin = 0 for the neuron, c = 1 for the SVM) performance is suboptimal for large numbers of patterns. There is an intermediate value of the training parameter (red, margin = 4 for the neuron, c = 10^− 6^ for the SVM) which gives optimal performance over a wide range of pattern numbers. Single neuron performance for the thresholds (0,1) (blue) is worse for all pattern numbers. Error bars show s.e.m for 25 different sets of random input patterns

Although differences were found in the precise margin-dependence of performance for different numbers of patterns, we found that a margin of M = 4 spikes allowed for optimal training in both cases. We next asked systematically how the performance of single-neuron classification depended on the number of patterns to be classified, for three different choices of the margin parameter. For comparison, we also evaluated the performance of a linear Support Vector Machine on the spike counts. SVMs are trained with a parameter *c* which weights the cost of misclassifying a pattern relative to the importance of providing a large margin. Figure 5 shows that the performance of single neurons trained with an optimal margin (*M* = 4, full red curve) closely tracks the performance of an SVM trained on the same inputs with an optimal *c* parameter (*c* = 10^− 6^, full black curve). Demanding too large of a margin for single neurons (*M* = 0, dotted red curve), or setting the SVM *c* parameter too low (*c* = 10^− 13^, dotted black curve) leads to poor performance specifically on easy tasks with low pattern numbers. Conversely, demanding too little of a margin for single neurons (*M* = 12, dashed red curve), or setting *c* too high (*c* = 1, dashed black curve) leads to a drop in performance for difficult tasks with large patterns numbers. We conclude that a margin of 4 provides good performance, close to that of a linear SVM, for a wide range of pattern numbers. Performance for the thresholds (0,1), which defines an algorithm similar to the voltage convolution implementation of the Tempotron rule (Gutig & Sompolinsky, 2006), is consistently worse for all numbers of patterns.

**Fig. 6:**
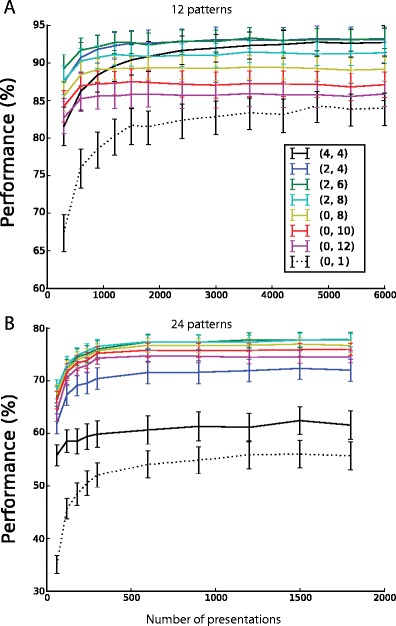
Learning speed increases as a function of the margin. For margin values ranging from 0 to 12, the evolution of the performance during learning is plotted for the thresholds giving highest asymptotic performance; for 12 patterns (panel (**a**)) and 24 patterns (panel (**b**)). We additionally plotted performance for the targets (0,1), which lead to both slower learning and lower asymptotic performance Error bars show s.e.m for 25 different sets of 12 or 24 random input patterns

In addition to affecting the asymptotic performance, the margin had a substantial effect on training speed. Figure 6a shows the evolution of performance throughout training, for various thresholds (θ-, θ+), when 12 patterns are learnt. For margin values ranging from 0 to 12, the pair of thresholds θ and θ + that is plotted is the pair giving highest asymptotic performance. We additionally plotted performance for the thresholds (0,1), which lead to both slower learning and lower asymptotic performance. Convergence to asymptotic performance is faster for larger margins. This may be understood intuitively: learning only occurs when there are mistakes, for example when the spike count to a + pattern does not exceed its learning threshold value. At the beginning of learning, this occurs more frequently if θ_+_  is high then if it is low. Likewise more mistakes occur at first if θ_−_ is low then if it is high. Learning is therefore fastest when the margin is large. Of the margin values that provide optimal performance in the classification of 12 patterns, a margin of 4 (which is also the optimal margin in a wide range of pattern numbers) thus provides the highest learning speed. For the classification of 24 patterns by single neurons, the influence of the margin on the convergence time is less evident, but the margin has a stronger effect on asymptotic performance (Fig. 6b).

**Fig. 7:**
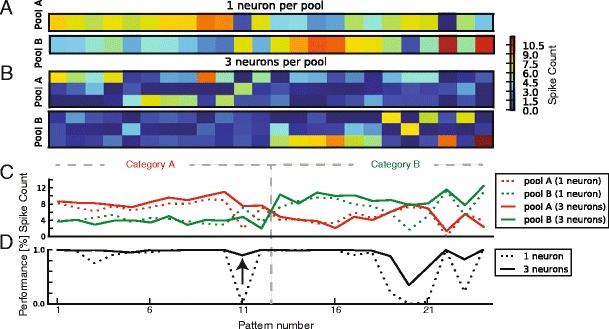
Multineuron pools outperform single neuron pools by evolving diverse receptive fields. A, (**b**), The mean responses of each neuron to each of the twelve (**a**) patterns (left) and each of the twelve (**b**) patterns (right) for single neuron (panel (**a**)) and for multineuron pools (3 neurons per pool) (panel (**b**)). **C**, Summed spike counts of pools (**a**) (red) and (**b**) (green). For single neuron pools (dashed lines) neuron (**b**) responds to the 11th A pattern whereas neuron (**a**) does not. For multineuron pools (full lines) this is reversed. **D**, Classification performance. Single neuron pools (dashed lines) completely misclassify the 11th (**a**) pattern, whereas multineuron pools (full lines) do not (arrow)

**Fig. 8:**
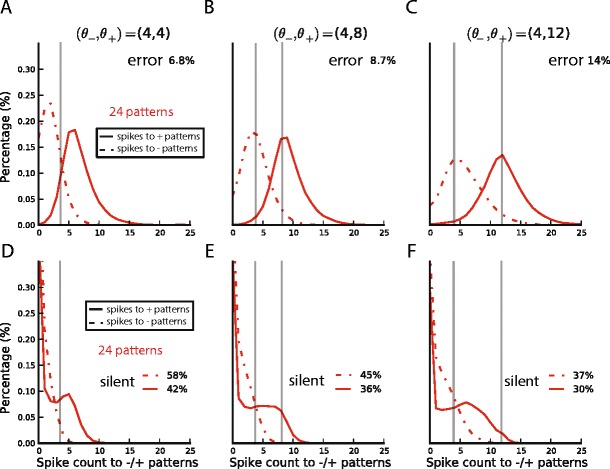
Multineuron pools implement sparse coding. Histograms of the summed number of spikes emitted by a pool (top row) versus by individual neurons (bottom row) to inputs from their category (full lines) and from the other category (dashed lines) for a training margin of 0 (left column (**a**), (**d**)), 4 (middle column (**b**), (**e**)) and 8 (right column (**c**), (**f**)). Insets in the top row indicate the pool’s classification error. Insets in the bottom row indicate the percentage of presentations to which an individual neuron is silent (the y-axis is truncated); this is much greater than the percentage of presentations to which the entire pool is silent (value at 0 of the histogram in the top row)

We next asked whether performance on nonlinearly separable tasks could be improved using multineuron pools, with the training signal depending on the summed number of spikes emitted by the pool. Multineuron pools were thus trained on the same highly linearly non-separable task which single neurons and the SVM perform with less than 80 % accuracy: the classification of 24 random rate patterns. All the neurons in a pool are trained with the same error signal, which depends on the number of spikes of the entire pool (see Materials and Methods for details). Although there was no lateral inhibition between the neurons, neurons in a given pool evolved different receptive fields. An example is shown in Fig. 7. The mean responses of each neuron to each of the twelve A patterns (left) and each of the twelve B patterns (right) is shown for single neurons (panel A) and for multineuron pools (3 neurons per pool) (panel B). To investigate how individual neurons shared the load of pool performance, we plotted spike count histograms of the entire pool (Fig. 8, top row) and of individual neurons from those pools (Fig. 8, bottom row). While the distribution of total pool spikes is again broad, individual neurons were silent in response to between 30 and 42 % of the + patterns (Figs. 8d and f). This indicates that the neurons have learned a sparse code, each having distinct receptive fields, which is the only way to solve this highly linearly non-separable task. This can be understood intuitively: since multineuron pools are simultaneously trained, if one of the neurons learns to respond to a pattern, then the pool receives no error signal, such that no other neuron in the pool needs to learn to respond to that pattern. The neurons then compete, in a “first-one-first-served” manner to learn the remaining patterns until all the patterns are learned.

**Fig. 9:**
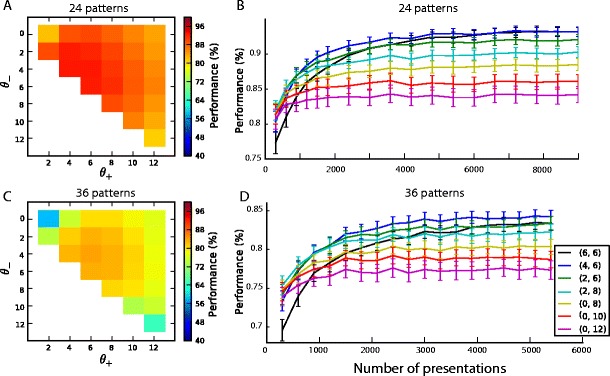
Performance and learning speed of multineuron pools as a function of the margin  M = θ_+_ − θ_−_. (**a**), (**c**) Performance as a function of the learning thresholds θ_+_ and θ_−_ for the classification of 24 (panel (**a**)) and 36 (panel (**c**)) input patterns: performance is highest for low values of the margin (near the diagonal). **B**, (**d**) For each margin value, the evolution of performance during learning is plotted for the thresholds giving highest asymptotic performance; for 24 patterns (panel (**b**)) and 36 patterns (panel (**d**)). Error bars show s.e.m for 25 different sets of 24 or 36 random input patterns. Learning is quickest for higher values of the margin

**Fig. 10:**
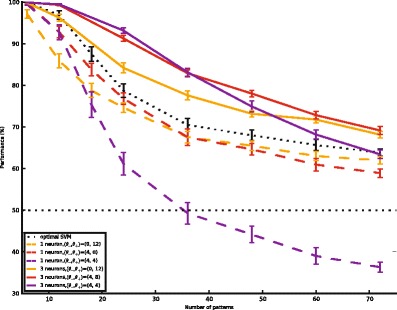
Comparison of single neuron and multineuron pools. Performance of 3 neuron (full lines) and single neuron pools (dashed lines) as a function of the number of patterns. For a low value of the margin (purple), performance is optimal for low pattern numbers. For a high value of the margin (yellow), performance is optimal for large pattern numbers. An intermediate value of the margin (red) provides optimal performance over a large range of pattern numbers. The optimal linear SVM performance is plotted in a dotted black line; note that this is exceeded by a 3-neuron pool. Error bars show s.e.m for 25 different sets of random input patterns

**Fig. 11:**
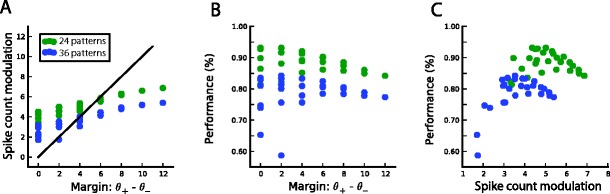
Relationship between training margin and actual spike count modulation for multineuron pools (defined as the difference between the mean spike count in response to target patterns and the mean spike count in response to null patterns). **A**, Scatter plot of the spike count modulation after learning as a function of the training margin. The actual modulation does not track the training margin, and is systematically larger for easier tasks (24 patterns in green versus 36 patterns in blue) (**b**), Scatterplot of the performance as a function of the training margin. **C** Same as in (**b**), but as a function of the spike count modulation

To investigate how margins affect performance when neurons are grouped in pools, we again systematically evaluated performance as a function of learning threshold values, for groups of 24 and 36 patterns (Figs. 9a and c). As with single neurons, increasing the margin increases training speed (Figs. 9b and d), but choosing margins too high impairs performance on easier tasks. A value of the training margin between 2 and 4 spikes provides good performance for these two tasks. Figure 10 shows performance over a range of pattern numbers, for three different choices of margin parameter; it can be seen that thresholds of (θ_−_, θ_+_) = (4,8) corresponding to a margin of 4 spikes again provide good performance over a wide range of linearly non-separable pattern numbers. In all cases, multineuron pools (full lines) provided improved performance over a linear SVM (dotted line). As in the single neuron case (dashed lines), we found that larger margins perform worse for easier problems, whereas small margins provide poorer performance for more challenging tasks with high pattern number. Also similarly to the single neuron cases, we found that performance depends more closely on the actual spike count modulation generated, rather than on the margin requested (see Fig. 11).

**Fig. 12:**
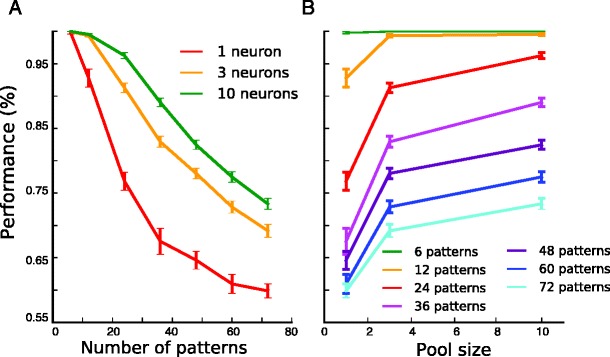
Capacity as a function of pool size. **A**, Performance for the optimal thresholds as a function of the number of patterns, for pools of 1 neuron (red), 3 neurons (yellow) and 10 neurons (green). **B**, Performance for the optimal thresholds as a function of the pool size, for various numbers of patterns. Error bars show s.e.m for 25 different sets of random input patterns

Finally, to investigate how capacity grows with the size of the pools, we plotted the performance for the optimal thresholds for pools of 1, 3 and 10 neurons (Fig. 12). By defining the capacity as the number of patterns for which mean performance is 90 %, one can read off from Fig. 12a that capacity increases from 14 for single neurons to 26 for 3 neurons and to 34 for 10 neurons. Similarly, Fig. 12b shows that increasing the number of neurons improves performance for each pattern number. The increases in performance for growing pool size are comparable to those found with reinforcement learning in populations of spiking neurons (Urbanczik & Senn, 2009).

## Discussion

In this study, we presented a learning rule which allows multineuron pools to learn in a supervised way to increase their firing rate in response to a certain set of inputs but not to another set. We combined an approach similar to the Tempotron (Gutig & Sompolinsky 2006; Gütig & Sompolinsky, 2009) for the synaptic update with concepts from the Support Vector Machine literature (Cortes & Vapnik, 1995). We found that a moderate training margin increases the learning speed of single neurons in linearly separable tasks, and increases their performance in linearly non-separable tasks. Although we did not assess the performance of the original Tempotron rule on our task, we found that using a (0,1) threshold a similar rule to the “voltage convolution” implementation of the Tempotron rule (Gutig & Sompolinsky 2006 produced worse performance on our task. We note however that the learning task originally used to test the Tempotron consisted of detecting reliable spatiotemporal patterns, whereas our task consists of discriminating Poisson spike trains that can vary from one repeat to the next. This may provide an explanation of the relatively poor performance of the (0,1) rule to some of the original applications of the Tempotron paper.

The performance of single neurons was bounded by the linear SVM performance, but performance could be increased by training neurons in pools with a single, global training signal. Although the neurons in a given pool received the same error signal derived from the pool’s number of spikes, they were nevertheless able to spontaneously select different features, thus classifying linearly non-separable inputs.

In models of unsupervised learning, lateral or recurrent inhibition is often used to force neurons to develop different receptive fields (Clopath et al., 2010; Masquelier et al., 2009; Yger & Harris, 2013). In the present case, recurrent inhibition was not necessary for neurons to evolve different receptive fields. Since our model has no feed forward inhibition, we normalised the rate patterns such that each pattern had the same global rate (otherwise, a pool would not be able to simultaneously respond with a high number of spikes to patterns of low input rate and with a low number of spikes to patterns of high input rate, and would therefore misclassify many patterns.) Adding divisive feedforward inhibition to the model might allow it to extend to the classification of non-normalised rate patterns. In the present model, synaptic weights were not allowed to become negative. Such a constraint typically reduces the capacity of perceptrons to learn rate-based inputs (see for example Amit et al., 1989; Gardner, 1988; Legenstein & Maass, 2007). This loss in capacity could be compensated for in part by adding subtractive feedforward inhibition to our model.

Could an analogous rule be implemented in the brain? The rule requires two steps: first, an eligibility trace is constructed based on pre-synaptic input occurring shortly prior to or during postsynaptic depolarization; and second, this is consolidated into a change in synaptic strength by a later-arriving training signal. Molecular mechanisms that could underlie the eligibility trace are well described, such as the multiple phosphorylation cascades that occur downstream of calcium influx via the NMDA receptor (Sweatt, 2009). But how might a training signal be conveyed? In the case of reinforcement learning, dopamine has been suggested as a training signal, and dopamine has indeed been implicated in the consolidation of eligibility traces (Kentros et al., 2004). A role for eligibility traces in reinforcement learning has been modelled previously (Izhikevich, 2007; Legenstein et al., 2008; El Boustani et al., 2012). A global reinforcement signal, however, cannot instruct different neuronal populations with different target signals. A more flexible, higher-dimensional training signal might instead be conveyed by glutamatergic inputs. In the cerebellum, for example, climbing fibre inputs provide strong inputs that generate complex-spike bursts which are believed to constitute a training signal (Eccles et al., 1967; Marr, 1969; Raymond et al., 1996). A second example consists of auditory fear conditioning, in which a conditioned reflex is established by the coincidence of signals conveying a conditioned stimulus (a tone) with a stronger unconditioned stimulus (a shock), by potentially glutamatergic inputs onto the amygdala (Pape & Pare, 2010). Understanding how spiking neurons may perform supervised learning at a computational level may lead to better understanding of such neuronal circuits.

## Electronic supplementary material

Below is the link to the electronic supplementary material.

Supplementary Figure 1Prediction of the firing rate from the membrane voltage. The graph shows ${\displaystyle \underset{0}{\overset{T}{\int }}}{\mathrm{F}}^2\left(\mathrm{V}\left(\mathrm{t}\right)\right)\mathrm{dt}$, used in the main text to predict the number of spikes emitted by a neuron (up to a multiplicative constant), as a function of the actual number of spikes emitted during a trial generated with the input statistics of the classification task. The multiplicative constant was fit with least squares. (PDF 960 kb)

Supplementary Figure 2 Number of input synapses and learning behaviour. The spikes for each component of the ten dimensional input rate patterns were spread out over 10 or 100 independent synapses (**a**), (**b**), Learning behaviour at the synaptic level (for an example synapse): the evolution of the weight of each of the duplicated synapses (grey) and their mean (green) is qualitatively and quantitatively similar to that of the original synapse (red). **C**, Learning behaviour at the neuronal level: the performance of single neurons with 100 input synapses tracks that of single neurons with 10 input synapses. Neurons were trained with the optimal thresholds (4,8), and the learning rate was increased according to the decrease in firing rate. Error bars show s.e.m for 25 different sets of random input patterns. (PDF 162 kb)
