# Supplementary figures and images for: Supervised learning with decision margins in pools of spiking neurons
Source: J Comput Neurosci. 2014 May 28;37(2):333–44. doi: 10.1007/s10827-014-0505-9 (PMC4159595; doi:10.1007/s10827-014-0505-9)

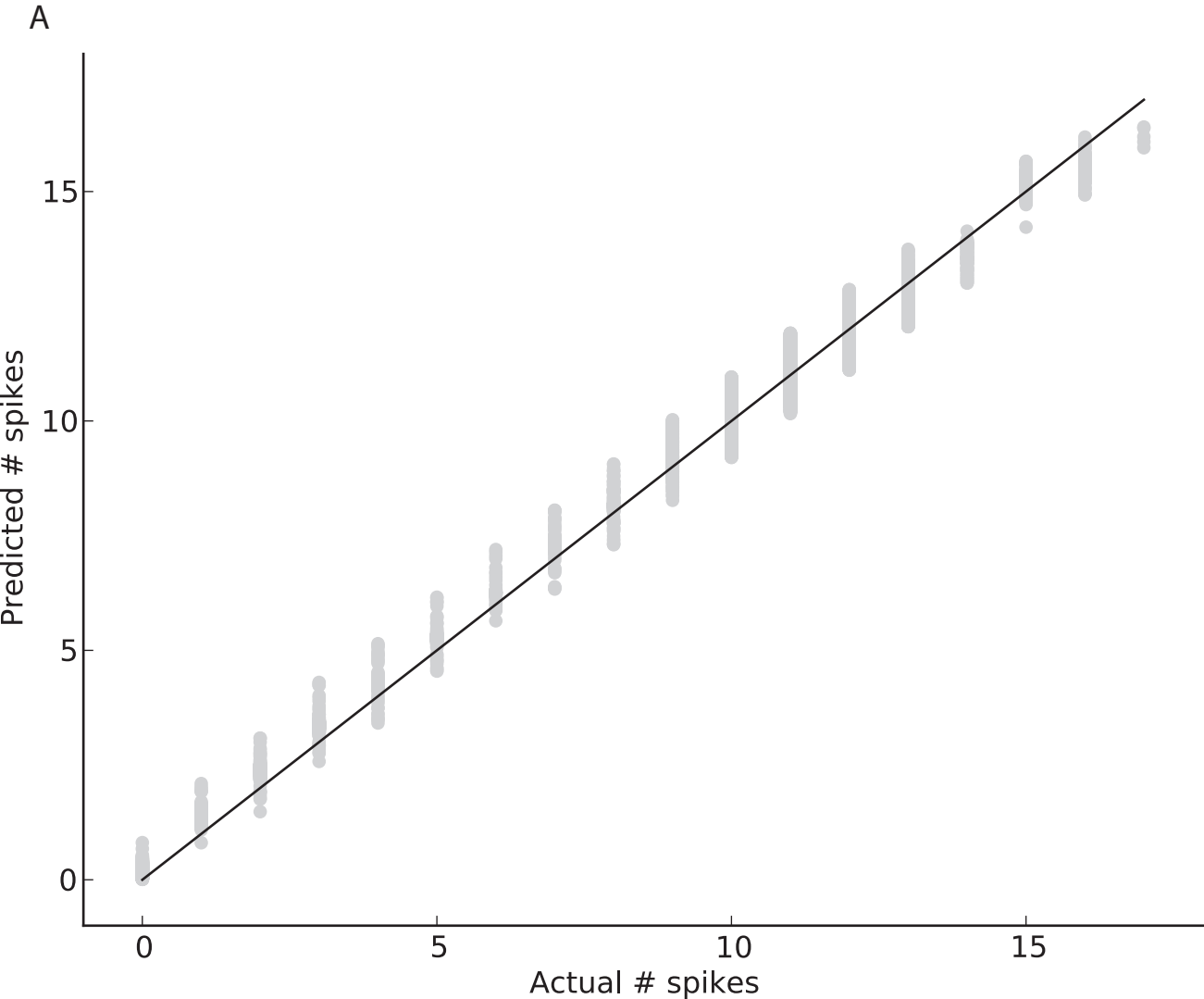

Supplement: Supplementary file 1 — Prediction of the firing rate from the membrane voltage. The graph shows ${\displaystyle \underset{0}{\overset{T}{\int }}}{\mathrm{F}}^2\left(\mathrm{V}\left(\mathrm{t}\right)\right)\mathrm{dt}$∫0TF2Vtdt, used in the main text to predict the number of spikes emitted by a neuron (up to a multiplicative constant), as a function of the actual number of spikes emitted during a trial generated with the input statistics of the classification task. The multiplicative constant was fit with least squares. (PDF 960 kb) [file 10827_2014_505_MOESM1_ESM.pdf]

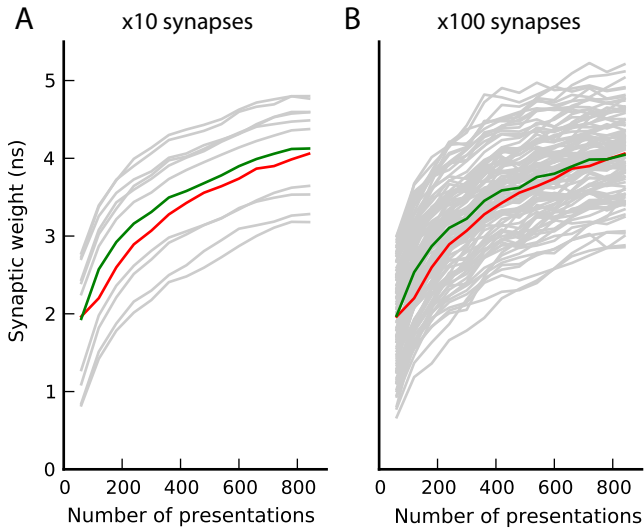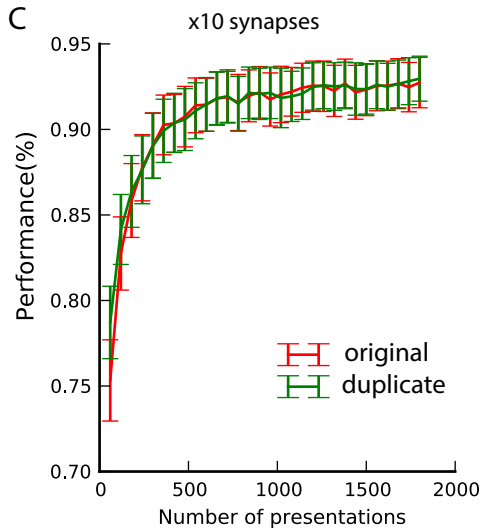

Supplement: Supplementary file 2 — Number of input synapses and learning behaviour. The spikes for each component of the ten dimensional input rate patterns were spread out over 10 or 100 independent synapses (a), (b), Learning behaviour at the synaptic level (for an example synapse): the evolution of the weight of each of the duplicated synapses (grey) and their mean (green) is qualitatively and quantitatively similar to that of the original synapse (red). C, Learning behaviour at the neuronal level: the performance of single neurons with 100 input synapses tracks that of single neurons with 10 input synapses. Neurons were trained with the optimal thresholds (4,8), and the learning rate was increased according to the decrease in firing rate. Error bars show s.e.m for 25 different sets of random input patterns. (PDF 162 kb) [file 10827_2014_505_MOESM2_ESM.pdf]
